# Chronic Pain and Chronic Opioid Use After Intensive Care Discharge – Is It Time to Change Practice?

**DOI:** 10.3389/fphar.2019.00023

**Published:** 2019-02-22

**Authors:** Dusica M. Stamenkovic, Helen Laycock, Menelaos Karanikolas, Nebojsa Gojko Ladjevic, Vojislava Neskovic, Carsten Bantel

**Affiliations:** ^1^Department of Anesthesiology and Intensive Care, Military Medical Academy, Belgrade, Serbia; ^2^Medical Faculty, University of Defense, Belgrade, Serbia; ^3^Imperial College London, Chelsea and Westminster Hospital NHS Foundation Trust, London, United Kingdom; ^4^Department of Anesthesiology, Washington University School of Medicine, St. Louis, MO, United States; ^5^Center for Anesthesia, Clinical Center of Serbia, Belgrade, Serbia; ^6^School of Medicine, University of Belgrade, Belgrade, Serbia; ^7^Universitätsklinik für Anästhesiologie, Intensivmedizin, Notfallmedizin, und Schmerztherapie, Universität Oldenburg, Klinikum Oldenburg, Oldenburg, Germany; ^8^Imperial College London, Chelsea and Westminster Hospital NHS Foundation Trust, London, United Kingdom

**Keywords:** critical care, pain, chronic pain, analgesics, opioids

## Abstract

Almost half of patients treated on intensive care unit (ICU) experience moderate to severe pain. Managing pain in the critically ill patient is challenging, as their pain is complex with multiple causes. Pharmacological treatment often focuses on opioids, and over a prolonged admission this can represent high cumulative doses which risk opioid dependence at discharge. Despite analgesia the incidence of chronic pain after treatment on ICU is high ranging from 33–73%. Measures need to be taken to prevent the transition from acute to chronic pain, whilst avoiding opioid overuse. This narrative review discusses preventive measures for the development of chronic pain in ICU patients. It considers a number of strategies that can be employed including non-opioid analgesics, regional analgesia, and non-pharmacological methods. We reason that individualized pain management plans should become the cornerstone for critically ill patients to facilitate physical and psychological well being after discharge from critical care and hospital.

## Introduction

The first intensive care units (ICU) were established in the United States during the 1960s, and since this time remarkable achievements have been made in survival rates of critically ill patients (Marini, 2015). However, prolonged periods of hospitalization in the ICU environment can impact significantly on patients’ overall wellbeing, at discharge from both ICU and hospital (Griffiths et al., 2013). Research regarding patient outcomes following ICU admission led to the development of the term “post-intensive care syndrome” (PICS). This term encompasses new or worsening physical, cognitive or mental health following a critical illness (Needham et al., 2012). This can include sleep deprivation, fatigue, weakness and chronic pain, alone or in combination and is common following hospital discharge after ICU treatment (Sukantarat et al., 2007; Timmers et al., 2011). It can be accompanied by anxiety, depression, post-traumatic stress disorder (PTSD) and deterioration in mental processing speed, memory, executive functioning and attentiveness (Sukantarat et al., 2007). These changes can persist up to 2 years following ICU discharge (Herridge et al., 2003, 2011). Therefore the development of chronic pain following ICU care as part of PICS or in isolation is an important patient outcome.

Most patients admitted to ICU receive opioids, commonly as part of analgesia and sedation regimens. A lack of adequate analgesia and acute pain can lead to chronic pain developing. Whilst ensuring patient comfort and alleviating pain is an essential aspect of patient care, the use of opioids for symptom control may not always represent an ideal approach. Opioid use can be associated with potential short and long term consequences, however, their development in patients after ICU discharge is not known. High cumulative doses or long term opioid use within critical care could lead to opioid dependence, tolerance, addiction, and physiological effects, such as hormone and immune system changes (Ballantyne and Mao, 2003). Tolerance occurs when there is a progressive decrease in the pharmacodynamic response to the drug and can be compensated by increasing the dose. This contrasts with dependence where a “physical effect, abstinence syndrome is seen upon abrupt drug withdrawal” (American Psychiatric Association, 2013). Addiction is a chronic disease, characterized by psychological dependence and an irreversible neurobiological disease with compulsive drug use (American Psychiatric Association, 2013). Opioid tolerance and/or dependence could be expected during and after ICU admission for patients who have received high or lengthy opioid dose regimens, however, there is little evidence regarding the incidence of these phenomena occurring and their impact on patients. The dichotomy then exists for critically ill patients of either having inadequate analgesia and developing chronic pain or having high doses of opioid analgesia and developing consequences such as dependence or long term use. The psychological and economical burdens of either of these are significant to patients, their families and society in general (Griffiths et al., 2013). It is therefore important to understand the reasons behind commencing opioid therapy for patients in ICU and determining whether it should continue at ICU discharge. This article aims to consider the emerging field of pain management within the ICU setting and outline possible management options to minimize both chronic pain and long term opioid use following ICU discharge.

## Methods

This narrative review aims to present the available evidence regarding both chronic pain and opioid dependence following ICU discharge. Additionally, it will review potential treatments and strategies to reduce the likelihood of patients developing chronic pain and opioid dependence following their ICU stay. The review focuses on adult patients. The search strategy was designed to find evidence which directly evaluated chronic pain. The terms used alone and in combinations were “chronic pain, critical illness,” “chronic pain, critical care,” “chronic pain, intensive care,” “opioids, critical illness,” “analgesics, opioids, critical care” “analgesics, non-narcotics, critical illness,” “analgesics, non-narcotics, critical care,” “sleep deprivation, critical illness,” “sleep deprivation, acute pain” in PubMed. The search period was for articles between January 1989 and August 2017. Articles were written in the English language and included those based on expert selection, open and blinded studies, reviews, meta-analysis, commentaries and editorials, related to the described MESH terms. From 5722 articles, we retrieved *n* = 184 articles based on the above criteria. In addition to the database search, we reviewed articles from reference sections in relevant articles to include additional articles not found by the original search. For analysis of chronic post ICU pain (CPIP) and chronic opioid use after ICU, articles were excluded if they didn’t clearly state in the methods that patients were treated in the ICU, that included pediatric patients, that did not clearly refer to chronic pain and chronic opioid use after ICU discharge. Nine articles were included for analysis of chronic pain after ICU (Granja et al., 2002; Boyle et al., 2004; Korošec Jagodič et al., 2006; Jenewein et al., 2009; Timmers et al., 2011; Battle et al., 2013; Griffiths et al., 2013; Choi et al., 2014; Baumbach et al., 2016) and one article for chronic opioid use (Yaffe et al., 2017).

### Chronic Pain After ICU

#### Definition

There is no widely accepted definition of chronic pain after ICU discharge (CPIP). Applying the definition for chronic pain used in the ICD 11 classification for the purpose of this review, we define chronic pain after ICU discharge as “pain persisting or recurring 3 months” after ICU discharge (Treede et al., 2015). There are no definitions for the type of pain (for example nociceptive, neuropathic or visceral), encompassed by CPIP and no studies included defined pain by type.

**Table 1:** Incidence of chronic post ICU pain.

Primary author and year	Type of study	Pain evaluation tool	Setting	Sample size	LOS (days/ hours)^∗^	VD (days/ hours)^∗^	HLOS (days/ hours)^∗^	Period of follow up (months)	Incidence of chronic pain	Pain location and intensity
Baumbach 2016	Case-control study (septic vs. non-septic) Single center	Validated German pain questionnaire	Germany Medical and surgical ICU	207	8.68 (9.17) days	69.09 (146.88) h	24.66 (21.32) days	6	33.2%	45% of those with pain reported intensity as moderate to severe
Battle 2013	Prospective for incidence Single center	Non-validated questionnaire Validated brief pain inventory for localization of body pain	United Kingdom (Wales) Medical and surgical ICU	196	6.2 days	2.1 days	17.8 days	6 and 12	44%	Commonest location was shoulder (22% of those with pain)
Boyle 2004	Prospective repeated measures observational study Single center	Pain Scale 10 point intensity-validated Pain self efficacy questionnaire-validated	Australia Medical and surgical ICU	*n* = 66 (1 month) *n* = 52 (6 months)	6.9 (5.5) days	57.1 (93.0) h	26.4 (30.2) days	1 and 6	47% 1 month 49% 6 months	Moderate to very severe pain 28% had pain more than half the days at 6 months
Choi 2014	Prospective longitudinal repeated measurement Single center	Modified given symptom assessment scale-not validated	United States Medical ICU	26	22.0 (10.2) days	18.9 (9.7) days	Not reported	4	53.8%	Mean pain intensity 5.4 on a 10 point scale
Granja 2002	Prospective cohort study Single center	EuroQol 5-D questionnaire	Portugal Medical and surgical ICU	275	2 days (range 1–120 days)	Not reported	Not reported	6	45%	Moderate to extreme pain
Griffiths 2013	Prospective Multicentre study	EuroQol-5D questionnaire (EQ 5D)-Validated EuroQol Visual analog scale-Validated Short form 36 Version 2-validated	United Kingdom Medical, surgical, trauma ICUs	293	8 (5–16) days	4 (2–11) days	29 (17–47) days	6 and 12	6 months-73% 12 months-70%	
Jagodic 2006	Prospective Two groups (sepsis and trauma) Single center	EuroQol-5D questionnaire-Validated	Slovenia Surgical ICU	39 (10 sepsis, 29 trauma)	11.4 (14.4) days	Not reported	40.0 (52.8) days	24	56%	
Jenewein 2009	Prospective Control group without CPIP Single center	Pain question asked by interviewer	Switzerland Trauma ICU	90	Not reported	Not reported	Not reported	36	44%	
Timmers 2011	Prospective observational cohort study Age- and gender-matched controls Single center	EuroQol-6D questionnaire (EQ 6D)-Validated	Netherlands Surgical ICU	575	5 (8) days	Not reported	19 (21) days	72–132	57%	Intensity VAS pain 69 (21) mm


*LOS, length of stay in ICU; VD, ventilator days; HLOS, hospital length of stay; PTDS, post-traumatic stress disorder. ^∗^Data are presented as mean (SD) or median (range).*

#### Incidence and Location

It is difficult to ascertain an exact incidence of CPIP. Nine articles reported incidence that varied widely between studies ranging from 33–73% (see Table 1). A variety of methods were used to evaluate CPIP between studies, which could account for these findings. Studies lacked consensus regarding the observation period in which chronic pain was evaluated. It ranged from 2 months to 11 years. Only one study considered pre-existing chronic pain, an important confounding factor (Baumbach et al., 2016). Other studies controlled for additional confounders such as age or gender. Study designs included comparisons to different control groups including septic vs. non-septic patients, ICU patients with and without CPIP, and age- and gender-matched individuals from the general population (Jenewein et al., 2009; Timmers et al., 2011; Baumbach et al., 2016). One study considered the bodily location of pain, which was found in approximately a fifth of patients at the shoulder (Battle et al., 2013).

**Table 2:** Risk factors for chronic post ICU pain.

Primary author and year	Patient population	Sample size	Risk factors identified
Battle 2013	Medical and surgical ICU	196	Increasing age and sepsis risk for chronic pain using multivariate analysis
Baumbach 2016	Medical and surgical ICU	207	Sepsis was not a risk factor for chronic pain
Boyle 2004	Medical and surgical ICU	52 (6 months)	Chronic pain patients had longer hospital LOS and longer time of ventilation
Granja 2002	Medical and surgical ICU	275	Surgical or trauma diagnosis associated with pain/discomfort using multiple logistic regression
Timmers 2011	Surgical ICU	575	Patient sex and trauma surgery independently associated with pain/discomfort


*LOS, length of stay.*

#### Risk Factors

Little is known about risk factors for developing chronic pain following ICU discharge. Five studies have attempted to explore these (see Table 2) considering the influence of ICU admission, ICU length of stay, duration of mechanical ventilation and duration of sepsis on the development of CPIP. Battle et al. (2013) identified an increased patient age and a diagnosis of “sepsis” as risk factors for CPIP. They further identified pain localised in the shoulder was influenced by “sepsis” and ICU length of stay (Battle et al., 2013). However, Baumbach et al. (2016) did not identify a diagnosis of sepsis as a risk factor for CPIP when accounting for the presence of persistent pain prior to ICU admission. Granja et al. (2002) found, however, that the main diagnosis of disease at ICU admission was a risk factor for developing pain and discomfort 6 months after ICU discharge, with patients admitted for trauma or surgery more likely to have CPIP. Both Timmers et al. (2011) and Baumbach et al. (2016) did not find ICU length of stay or days of mechanical ventilation influenced the development of CPIP. Although CPIP was evaluated using verbal descriptors or visual analogue scales (VAS) for pain intensity in four studies, pain intensity was rarely considered in studies, rather presence or absence of pain. As such, it is impossible to conclude if acute pain intensity whilst in ICU influences the development of CPIP (Griffiths et al., 2013; Choi et al., 2014). Therefore there is a paucity of evidence regarding predisposing factors to developing CPIP.

Observational data from Europe regarding the development of chronic post surgical pain (CPSP) highlights preexisting persistent pain as being associated with a 2.6 times higher risk of developing CPSP (Fletcher et al., 2015). Moreover, the percentage of time in severe pain on day one post surgery was found to be predictive of developing CPSP (Fletcher et al., 2015). Unfortunately there is a paucity of evidence to support this finding with respect to CPIP, as only one study considered this and did not identify it as an independent risk factor (Choi et al., 2014). Kemp et al. (2017) showed that 35.5% of ICU patients had a doctor assess pain and few used validated pain assessment tools. If healthcare professionals fail to assess pain, not only does this mean management may not be optimal, but using the analogy of CPSP, untreated acute pain in ICU could predispose to developing CPIP. Studies investigating the connection between CPIP and pain experienced by patients whilst in ICU are therefore needed.

#### Consequences of CPIP

Unsurprising, CPIP affected the physical, psychological and social well-being of patients. CPIP was found to compromise normal everyday living including normal work, walking, relationships with friends and family, activity, mood and sleep (Baumbach et al., 2016; Puntillo and Naidu, 2016). Baumbach et al. (2016) found that 60% of patients who developed CPIP found this moderately to severely interfered with their daily life, family activities and work. Furthermore family members who care for ICU survivors with moderate and severe pain demonstrated higher distress scores themselves (Griffiths et al., 2013). Chronic pain in general is “highly comorbid with anxiety and depression” and whilst this might also be the case for CPIP, no studies found this association (Katz et al., 2015). CPIP was found to be associated with PTSD (Jenewein et al., 2009). Whilst severe accidental injury requiring ICU admission is likely to lead to physical disability it is notable that in a prospective follow up study of ICU survivors who experienced severe accidental injuries, those with CPIP had a significantly more frequent presence of physical disability, occupational invalidity and absence from work than those pain free up to 3 years following their injury (Jenewein et al., 2009). Overall these data represent the high burden CPIP places on ICU survivors.

With the limited information available we can conclude that the incidence of CPIP is high, however, there is conflicting evidence supporting predisposing risk factors to its development. Bodily localization, pain intensity and type of pain CPIP encompasses are rarely investigated, and there was no evidence to consider the link between pain experienced during a patients ICU stay and the development of CPIP.

### Chronic Opioid Use After ICU Discharge

Intensive care unit patients risk continuing opioids following ICU discharge and potentially after leaving hospital due to management of CPIP. Chou et al. (2009) adapted Von Korff et al. (2008) definition of chronic opioid therapy as “daily or near-daily use of opioids for at least 90 days, often indefinitely.” One study was identified that considered opioid use following ICU discharge. Yaffe et al. (2017) investigated opioid use in ICU survivors with surgical and non-surgical diagnoses by retrospectively analyzing electronic patient charts. They identified opioid use in 12.2% of patients at hospital discharge. This proportion fell to 4.4% when re-evaluated 48 months later. No difference was found regarding chronic opioid use between medical and surgical patients. There was a “discrepancy” noticed by the authors between reported high incidence of CPIP and low chronic opioid use. This was hypothesized to reflect non-opioid pain management, a change in opioid prescribing for non-cancer pain and the lack of adequate tools to define chronic pain syndromes. However, chronic opioid use prior to ICU admission and length of hospital stay were associated with post-discharge chronic opioid use (Yaffe et al., 2017). The study, however, did not evaluate consequences of long-term opioid use or the intensity and type of pain experienced by chronic opioid users.

It is obvious that moderate and severe acute pain experienced by patients in ICU requires opioid therapy; however, a balance is required to ensure patients are comfortable throughout their ICU admission without predisposing them to require opioids long term after ICU discharge. Certainly, further exploration into the incidence and consequence of chronic opioid use following ICU admission is important especially in light of current concerns regarding long term opioid use in general.

### Preventive Measures

There is little evidence to support specific risk factors in developing CPIP, however, preventative measures should in the first instance be extrapolated from CPSP literature that supports managing acute pain to reduce the risk of transition to chronic pain states. There are few papers considering interventions targeted at acute pain management within the ICU setting and altered critical care outcomes. Evidence suggests, however, that simply performing regular validated pain assessments is associated with improved patient outcomes (Payen et al., 2009; Skrobik et al., 2010). Below pharmacological and non-pharmacological measures are explored that consider predicting those at risk in the pre ICU stage, managing acute pain within the ICU admission and management following discharge which could reduce the transition from acute pain to CPIP.

#### Pre-ICU Admission: Assessment of Risk Factors

Detailed analysis of a patients’ preadmission state might play a significant role in identifying those at risk of developing CPIP. Patients with increasing age, and a diagnosis at ICU of trauma, or surgery should be considered as high risk. Additionally, evidence from CPSP suggests a more pre-emptive management strategy including a pre-emptive analgesia model using antidepressants or anticonvulsants early during the ICU stay could influence the development of CPIP. Utilizing both pain focused psychological management and physiotherapy early in ICU admission could also reduce risk. Furthermore, a history of opioid use or misuse prior to admission should lead to careful use of opioids during admission.

### During ICU Admission

Each patient should have an individualized pain management plan. This should account for the leading health problem and comorbidities, avoid medications that could deteriorate the patients critically ill state and aim to cover pain management throughout the patient’s ICU stay. This plan should be flexible to account for changes in treatment and clinical condition. The ABCDEF Bundle is an evidence based flexible framework that is a multimodal and multidisciplinary, patient and family centered approach for the management of the critically ill patient. “A” within the bundle represents “Assessment, Prevention, and Management of Pain” (Ely, 2017). This is a useful starting point for the pain management plan.

The American College of Critical Care Medicine (ACCM) recommends regular and meticulous assessment of pain and sedation, followed by careful titration of opioids and sedatives (Barr et al., 2013; Devlin et al., 2018) and prevention and treatment of procedural pain (Barr et al., 2013). Targeting analgesic doses to patients by using pain assessment ensures individualized treatment and is associated with a reduction of analgesic consumption. Pain assessment should ideally use self reported scales such as the numeric rating scale (Chanques et al., 2010), however, this can be limited by functional disability from critical illness. It is imperative to ensure pain is evaluated by an alternative method in the non-communicative patient (Laycock and Bantel, 2016). Here, the ACCM recommend using The Behavioral Pain Scale (BPS) or the Critical-Care Pain Observation Tool (CPOT) (Barr et al., 2013; Devlin et al., 2018). Whilst non-subjective measures of pain have been developed which include new objective tools based on physiological variables including electroencephalography, flexion reflex, and pupillometry, their utility in the critically ill has been conflicting. The analgesia nociceptive index, using heart rate variability as response of the autonomic nervous system, showed no correlation with BPS (Broucqsault-Dédrie et al., 2016). Pupillometry was used for pain measurement during endotracheal suctioning, but pupil size is influenced by multiple factors which could confound the response (Paulus et al., 2013). Nociception flexion reflex (NFR) threshold is based on scoring a patient’s protective withdrawal reflex (Skljarevski and Ramadan, 2002) and although results correlate with self-reported pain, the lack of a standardized scoring system limits its clinical application (Cowen et al., 2015). A biological marker that measures the genetic response through protein synthesis represents an ideal objective method of measuring pain, however, nociception is complex. It involves neuroendocrine and inflammation responses which affect multiple systems. In this respect a combination of several biological markers could represent the panacea of pain assessment in the critically unwell (Cowen et al., 2015). Until this is developed self report and behavioral tools mentioned are the most evidence based methods to ensure targeting analgesia to evaluate efficacy.

#### Pharmacological interventions

Pain should represent the principle tenant of analgosedation management in critical care. Pain and discomfort is recommended to be addressed prior to sedative use and escalation. This approach has been shown to shorten length of mechanical ventilation and ICU stay (Devabhakthuni et al., 2012). Using the analogy with post-operative pain where multimodal analgesia (MMA) is widely accepted, in the critically ill patient this principle can provide an opioid sparing effect and better quality of analgesia (Payen et al., 2013; Wick et al., 2017). One of the rare studies investigating MMA in ICU, found that MMA reduced doses of sedatives when used for baseline pain management, procedural pain, pain related to disease, and discomfort caused with mechanical ventilation (Payen et al., 2013). The authors suggest that MMA concept can be applied to a wider number of critically unwell patients not just for patients with lower illness severity scores and ability to self rate their pain intensity (Payen et al., 2013). However, its application can be challenging in the critically unwell as multi organ insufficiency, preexisting comorbidities and polypharmacy can hinder drug choice and combinations. As physiologically unwell patients present with variable pharmacodynamics and kinetics, frequency, severity and types of pain conditions, it is suggested that methods of dosing medications should be individualized, as well as mode of intravenous delivery (intermittent vs. continuous), and route (oral, intravenous, or through the catheters for epidural or regional blocks) (Barr et al., 2013).

##### Opioids

Intravenous opioids are the mainstay of treating moderate to severe non-neuropathic pain in ICU (Barr et al., 2013). However, their side effect profile includes immunosuppression, hypotension, respiratory depression, chest wall rigidity and gastro-enteric dysmotility (Devabhakthuni et al., 2012). Some side effects can be dose dependent such chest wall muscle rigidity. Choice of opioid remains controversial as whilst remifentanil pharmacokinetic profile, with rapid offset and minimal drug accumulation, has advantages over fentanyl and morphine (Devabhakthuni et al., 2012), prolonged use can lead to tolerance and opioid induced hyperalgesia (Pandharipande and Ely, 2005).

A history of opioid use or misuse prior to critical care admission presents a challenge. As with surgical patients on chronic opioids, normal pre-admission opioid doses should be continued as a baseline should the patients’ physiology allow it. Additional short acting opioids, ideally as patient controlled analgesia, can be used for additional pain needs alongside other multimodal strategies. Where oral administration of medications is not possible, pre-admission oral medications should be changed to intravenous forms (Peng et al., 2005; Alford et al., 2006; Mehta and Langford, 2006). Prior to ICU discharge, there should be carefull tapering of opioids where appropriate to ensure long term opioids are not continued unnecessarily leading to chronic opioid use (Azzam and Alam, 2013). The aim should be to return patients to at most the doses they were taking prior to critical care admission. Iatrogenic withdrawal syndrome (IWS) caused by abrupt tapering of opioids in adult ICU patients is rarely explored in the literature. Small cohort studies recorded IWS in 16–32% opioid treated adult ICU patients (Cammarano et al., 1998; Wang P.P. et al., 2017). Wang P.P. et al. (2017) identified risk factors including a higher cumulative opioid dose and longer time of opioid use. There are no studies considering how best to taper opioids in the critically ill adult. Consensus guidelines from pediatric intensive care where tapering of opioids and sedatives is common place, suggest no specific regimen, however, one could consider reducing 5–10% of daily dose every 24 h (Playfor et al., 2006). Other potential options to reduce the risk of IWS include the use of methadone, alpha-2 agonists or NMDA receptor antagonists alone or in conjunction during opioid reduction (Puntillo and Naidu, 2016).

##### Non-opioid analgesics

Non-opioid analgesics are recommended for use in ICU to reduce or terminate use of opioids and therefore reduce opioid side effects (Barr et al., 2013). Paracetamol and non-steroidal anti-inflammatory drugs (NSAIDs) safety profile and effectiveness are rarely and incompletely studied in critically ill patients (Elia et al., 2005; Barr et al., 2013). NSAIDs inhibit cyclooxygenase (COX) enzyme and this result in prostaglandin production reduction. COX-2 inhibitors are developed for selective inhibition of COX-2 enzyme isoform and show reduced risk for gastric bleeding compared to NSAIDs. Both are associated with numerous side effects, the most important of which in the critically ill are risks associated with renal insufficiency, cardiac failure and bleeding (Curtis et al., 2004). The antipyretic and analgesic effects of paracetamol are in part explained by selective COX inhibition within the central nervous system, and also indirect effects via the vanilloid subtype one receptor and cannabinoid type one receptors in the central nervous system (Bertolini et al., 2006). Whilst concerns regarding hepatic insufficiency and toxicity in the critically ill are likely minimal if dosing is carefully considered, recent evidence regarding possible hypotension caused by paracetamol and its risk in patients with instability requires consideration prior to use (Weinberg et al., 2015). There is a paucity of evidence regarding the role of NSAIDs, COX-2 inhibitors and paracetamol, as part of MMA regimen in critically ill patients, with focus on “clinically relevant” dynamic pain intensity reduction, reduction of opioid side effects and presence or absence of their own side effects (Elia et al., 2005). Whilst literature from the post-operative pain setting supports their inclusion especially as opioid sparing agents their application in the ICU setting remains controversial and should be considered on an individual patient basis (Remy et al., 2005; Mcdaid et al., 2010; Jefferies et al., 2012; Barr et al., 2013).

##### Local anesthetics

Local anesthetics can be used in regional anesthesia techniques or as an intravenous infusion (lidocaine). Regional analgesia remains controversial in the critically ill. The benefits include cardioprotective effects, especially from thoracic epidural analgesia, reduced risk for deep venous thrombosis, opioid sparing and improved analgesia that aids tracheal extubation (Liu et al., 1995; Carrier et al., 2009; Bignami et al., 2010; Horlocker et al., 2010; Caputo et al., 2011). Furthermore catheter placement enables continuous peripheral nerve blockade with local anesthetics in low concentrations (Williams et al., 2003; Casati et al., 2007; Bernards et al., 2008). However, they are not without risks which include infection and bleeding in the presence of critical illness associated coagulopathy (Hebl, 2006; Hebl and Neal, 2006; Horlocker and Wedel, 2006; Wedel and Horlocker, 2006; Horlocker et al., 2010). ACCM guidelines recommend thoracic epidural anesthesia/analgesia consideration for post-operative analgesia in patients undergoing abdominal aortic aneurysm surgery and for patients with traumatic rib fractures (Barr et al., 2013). However, due to lack of evidence no recommendation was given for neuraxial/regional analgesia over systemic analgesia in medical ICU patients (Barr et al., 2013). Therefore regional analgesia application in the critically ill should be individually evaluated.

There is no data regarding the use of intravenous lidocaine for analgesia in critically ill patients. Perioperative intravenous infusions of lidocaine are recommended for colorectal surgery by the PROSPECT Working Group (2017). This has been shown to reduce pain scores and post-operative opioid consumption by 85% in various surgical procedures and to reduce length of hospital stay (McCarthy et al., 2010), however, more recent evaluation of the evidence is less convincing (Kranke et al., 2015). Running lidocaine intravenous infusions needs to be considered in light of potential side effects pertinent in the critically unwell, including cardiac arrhythmia and accumulation secondary to altered metabolism in those with heptic or renal failure (Dickerson and Apfelbaum, 2014).

##### Co-analgesics

Ketamine is a non-competitive NMDA receptor antagonist, with the potential to prevent central sensitization, wind up phenomenon and opioid induced tolerance (Woolf and Thompson, 1991; Mao et al., 1995). It is used to reduce post-operative pain intensity, is opioid sparing (Laskowski et al., 2011) and has demonstrated beneficial antidepressive and neuroprotective effects (Hudetz and Pagel, 2010; Kishimoto et al., 2016). Ketamine could be considered in pain refractory to other therapies in the critically unwell (Patanwala et al., 2017). However, there is also concern that extrapolating from evidence in elderly post-operative patients where ketamine was associated with negative experiences including hallucinations and nightmares (Avidan et al., 2017), its influence on ICU delirium requires attention prior to application. Additionally its cardiovascular effects mean use should be considered on an individual basis (Erstad and Patanwala, 2016).

Alpha-2 agonists provide analgesia, anxiolysis, have sedative effects and do not cause respiratory depression (Chen et al., 2015; Gagnon et al., 2015). Dexmedetomidine has been shown to reduce the duration of mechanical ventilation and ICU stay compared to “traditional sedatives” (Chen et al., 2015). It can be used as a sole analgesic or with opioids in critically ill patients (Shehabi et al., 2004). In experimental studies, alpha-2 adrenergic agonists demonstrated antinociceptive effects in visceral pain conditions (Ulger et al., 2009). Furthermore dexmedetomidine has been shown to prolong the duration of sensory block provided by local anesthetics during regional analgesia (Akin et al., 2008; Kang et al., 2018). Its application is promising although associated bradycardia and hypotension need to be considered in the critically unwell (Devabhakthuni et al., 2012). Clonidine, another alpha-2 agonist, represents an alternative drug that again has demonstrated opioid-sparing effects; however, it has cardiovascular effects including hypotension and is reported to induce a withdrawal syndrome (Wang J.G. et al., 2017). Its effect on sedation level in ICU has been investigated with the conclusion that it can be an effective and safe alternative to a dexmedetomidine infusion, but its effect on CPIP prevention has not been considered (Terry et al., 2015).

Gabapentinoids, gabapentin and pregabalin, are anticonvulsants recommended for use in neuropathic pain management (Finnerup et al., 2015). They share the main mechanism of action, via α2-δ subunits of voltage-sensitive calcium channels in presynaptic afferent neurons (Quintero, 2017). Gabapentoids can decrease analgesia requirements and sedative medication use, but they are rarely investigated in adult critically ill patients, despite the promising results from pediatric ICU (Pandey et al., 2005; Sacha et al., 2017). Beside sedation, dizziness, visual disturbances as adverse effects, respiratory depression was described with gabapentin and opioid combinations and concern is now present as they potentially represent drugs of abuse (Cavalcante et al., 2017). Pregabalin can cause confusion, somnolence, potentiate respiratory depression of remifentanil and adverse effects on cognition and addiction (Myhre et al., 2016; Bonnet and Scherbaum, 2017). Based on all the above, and with no intravenous formulation, there is question of clinical usefulness of gabapentoids in the critically unwell.

Certain antidepressants are effective in neuropathic pain management, however, their ability to prevent developing chronic pain states is unknown (Saarto and Wiffen, 2007). Whilst amitriptyline and drugs such as duloxetine show promise in chronic pain states, their application in critical care has yet to be explored. Side effect profiles such as cardiac effects of amitriptyline and drug interactions mean their use in the critically unwell may be limited and lacks clinical evidence. Serotonin-norepinephrine reuptake inhibitors (SNRI) are recommended as first line treatment in neuropathic pain (Finnerup et al., 2015). However, their effect on hemodynamics and liver function limits their clinical usefulness in critically unwell patients and they are not recommended for use in current pain guidelines for critically unwell patients with neuropathic pain (Devlin et al., 2018).

##### Other medications

Glucocorticosteroids mechanistically should produce analgesia. They have multiple actions including inhibition of prostaglandin synthesis, influence on activity and plasticity of the nervous system and reduction of spontaneous discharge in injured nerves (Watanabe and Bruera, 1994; Mensah-Nyagan et al., 2009). Furthermore, endogenous neurosteroids control various mechanisms involved in pain sensation and modulate GABA, NMDA, and P2X receptors (Mensah-Nyagan et al., 2009). However, glucocorticosteroids have numerous side effects, and a case controlled study found an increased rate of infection, increased ICU stay and increased mechanical ventilation, with a trend toward increased mortality in critically ill treated with steroids (Britt et al., 2006). Despite studies in post-operative analgesia where dexamethasone has been shown to reduce post-operative pain and opioid consumption, there are no data available regarding the benefits of glucocorticosteroid use as adjuvants for pain management in critically ill patients (De Oliveira et al., 2011).

There is a relationship between sleep and both chronic and acute pain (Finan et al., 2013). Disturbed and shortened sleep has been shown experimentally in healthy volunteers to cause hyperalgesia modification to pain perception and increase reported pain intensity (Roehrs et al., 2006; Azevedo et al., 2011), and the descending pain modulatory system is suggested as a mediator for sleep disruption modulated hyperalgesia (Tiede et al., 2010). ACCM recommends “promoting sleep in adult ICU patients by optimizing patients’ environments, using strategies to control light and noise, clustering patient care activities, and decreasing stimuli at night to protect patients’ sleep cycles.” Additionally the use of melatonin has been suggested in a recently published summary of the Future of Critical Care Medicine meeting (Barr et al., 2013; Marini et al., 2017). Individual patient pharmacokinetics of melatonin are extremely variable, and in critically unwell patients earlier peak, greater plasma concentration and slower plasma clearance have been described and therefore doses should be reduced to accomodate for this (Bellapart and Boots, 2012). Additionally low clearance can lead to potentially “nocturnal” plasma levels during the late morning that may nullify potential “chronotherapeutic” benefits of melatonin (Arendt and Skene, 2005; Bourne et al., 2008). It has been reported that to reach sufficient sleep length and sleep quality, in some cases melatonin needs to be given for up to 3 days, as a result of progressive melatonin sensitization and an effect on timing mechanisms of sleep (Arendt et al., 1984; Boyoko et al., 2012). The dose, formulation, timing of administration, duration of melatonin treatment and assessment of optimal circadian timing are still unclear in the critically unwell (Arendt and Skene, 2005).

There is no evidence regarding the interaction between sleep in ICU and either the experience of acute pain or the development of CPIP, however, it is possible that addressing sleep by prescribing melatonin, adjusting lights in order to imitate day/night pattern, and providing windowed rooms can improve circadian rhythm and sleep which in turn could influence pain (Madrid-Navarro et al., 2015; Mo et al., 2016).

#### Non-pharmacological methods

Non-pharmacological methods used as part of MMA regimen in the critically unwell might increase the effect of medications, reduce opioid consumption and incidence of adverse drug events, and reduce the need for opioids post-discharge.

##### Psychological support

Critical illness subjects patients to psychological stress, anxiety, low mood, fear of dying and hallucinations (Novaes et al., 1999; Jones et al., 2007; Wade et al., 2013; Hadjibalassi et al., 2018). This can be attributed to the experience of regular life disruption, the physical ICU environment, an inability to communicate, and the effects of critical illness and invasive treatments such as mechanical ventilation. This can be associated with long term psychological consequences including anxiety, depression, PTSD and chronic fatigue syndrome (Griffiths et al., 2004). PTSD can accompany chronic pain following critical illness, and whilst the exact association is unknown, psychological interventions for PTSD could impact on CPIP (Jenewein et al., 2009). It is important to note PTSD interventions are most effective when implemented early rather than post-discharge (Hatch et al., 2011; Wade et al., 2013). This also emphasizes the importance of a clinical psychologists being part of the ICU multidisciplinary team (Hatch et al., 2011; Peris et al., 2011). It is also important to have excellent communication between the multidisciplinary team, the patient and their relatives (Meraner and Sperner-Unterweger, 2016). Nursing staff have an essential role in supporting patients psychologically including explaining interventions and establishing a relationship with both the patient and their family (Pattison, 2005; Clancy et al., 2015; Marini, 2015).

##### Virtual reality

Virtual reality (VR) is computer-generated simulation of a 3D image delivered with special equipment that can interact with a patient’s pain by distraction (Hoffman et al., 2011). It can have an opioid sparing effect and be associated with pain reduction in burn patients, especially during wound care and physical rehabilitation (Carrougher et al., 2009; McSherry et al., 2018). In critically ill patients VR neurocognitive based therapy improved attention and memory (Turon et al., 2017) and it has a role in reducing opioid abuse in chronic pain patients and opioid dependence in heroin addicts (Gupta et al., 2018). Certainly VR looks promising as an additional strategy in managing pain in ICU patients, however, further evidence including patient selection and randomized controlled trials to evaluate effects of VR on different types of procedural pain and overall pain states is warranted (Hoffman et al., 2011; Indovina et al., 2018).

##### Music therapy

Music therapy is defined by the American Music Therapy Association “as the clinical and evidence-based use of music interventions to accomplish individualized goals within a therapeutic relationship by a credentialed professional who has completed an approved music therapy program” (American Music Therapy Association [AMTA], 2011). Music is a cognitive distractor (Chlan et al., 2013; Birnie et al., 2017), switching attention via the thalamus to the prefrontal cortex rather than painfull input and activating the endogenous opioid system to modulate perception of pain (Economidou et al., 2012). Positive effects of music therapy in ICU patients can be summarized as anxiety reduction, improvement of blood pressure, heart, and respiratory rate, quality of sleep and reduced pain intensity and analgesics requirement (Bradt et al., 2013; Hole et al., 2015). Meta analysis of 14 randomized trials focused on music therapy effects in ICU patients on mechanical ventilation and showed reduced anxiety, respiratory rate, systolic blood pressure and use of sedation and analgesia medications (Bradt and Dileo, 2014).

##### Physiotherapy

Physiotherapy could prevent joint and limb pain, particularly by reducing joint contractures, important in CPIP (Clavet et al., 2008; Battle et al., 2013). Muscle atrophy and weakness caused by prolonged bed rest during ICU treatment can reduce patient mobility which persists up to 2 years after discharge (Fan, 2012; Connolly et al., 2015). No data regarding the influence of early physical therapy on CPIP are currently available, however, this also warrants further investigation.

#### Post-discharge Period

##### Pain follow up clinic

Follow up to evaluate CPIP is important following ICU discharge. This allows for evaluation of intensity and nature of continued pain, to ensure appropriate analgesic prescribing and the development of a long term plan for pain management. It is likely this should occur within a specialized pain clinic that would ensure psychological assistance. A neurorehabilitation based protocol including use of patient diaries, focus groups with other ICU survivors, cognitive and physical rehabilitation may be useful to encorporate in such services (Clancy et al., 2015). This comprehensive follow up in a dedicated service is essential due to the significant number of patients that have pain despite multimodal and multidisciplinary approaches to its management (Lasiter et al., 2016; Hållstam et al., 2017).

## Future Trends

Genes code proteins with different roles, including receptors, enzymes, proteins responsible for substance transfers and biometabolic mediators. Analysis of genetic polymorphism can identify responders to particular medications and patients response to treatment, providing personalized pain management in the intensive care (Celi et al., 2013; Papaioannou et al., 2014; Marini et al., 2015). In some critical illness states molecular assays and gene mapping are already the reality of treatment. As previously highlighted with respect to pain assessment, however, it is likely that a panel of biomarkers is more appropriate for evaluating pain, rather than one specific biomarker (Bäckryd, 2015).

## Conclusion

Modern pain management on ICU should not only address acute pain, but also aim to prevent the development of CPIP and ongoing opioid use. It’s management should involve a protocolized approach that can be adjusted for each patient to account for comorbidities and their presenting complaint. This needs a systematic individualized multimodal pharmacological and non-pharmacological treatment approach that utilizes a multidisciplinary team. So far, no single medication or technique has been proven as most effective in either acute management within ICU or as a preventative measure for the development of CPIP. However, strategies should include regular detailed pain assessments using appropriate tools to evaluate initial pain and response to treatment. Pharmacological strategies should involve the lowest possible dose of opioids that is still effective, with the aim of the earlist possible decline of the dose and slow transition to non-opioid medications.

However, before individualized approaches can be fully developed and evaluated there needs to be research that leads to further understanding of risk factors for CPIP and evaluate whether long term opioid use is prevalent in this population. Answering these important questions could help guide management to ensure the long term consequences of either chronic pain or opioid dependence are not experienced in the future by ICU patients.

## Author Contributions

DS initiated this report, designed the work, collected the data, interpreted the data, and wrote the manuscript. HL designed the work, collected the data, interpreted the data, wrote and revised the manuscript. MK, NL, and VN contributed to literature search and collected data. CB designed the work, collected the data, interpreted the data, and revised the manuscript. All authors read and approved the final manuscript.

## Conflict of Interest Statement

The authors declare that the research was conducted in the absence of any commercial or financial relationships that could be construed as a potential conflict of interest.
